# Mortality risk on farm and during transport: a comparison of 2 broiler hybrids with different growth rates

**DOI:** 10.1016/j.psj.2023.103395

**Published:** 2023-12-23

**Authors:** Merete Forseth, Randi O. Moe, Käthe Kittelsen, Ingrid Toftaker

**Affiliations:** ⁎ Norsk Kylling AS, 7300 Orkanger, Norway; † Faculty of Veterinary Medicine, Norwegian University of Life Sciences, 1433 Ås, Norway; ‡ Animalia, Norwegian Meat and Poultry Research Centre, 0513 Oslo, Norway

**Keywords:** slower-growing, broiler, mortality, transport, welfare

## Abstract

Mortality in broilers is often associated with poor health and welfare and has a complex and multifactorial etiology. Recent studies under experimental conditions indicated that genetic selection for fast growth is an important risk factor for mortality in broiler chickens. However, the knowledge on broiler mortality in general, and in slower growing broilers in particular, under commercial conditions is still limited. This retrospective cohort study aimed to describe mortality risk on farm and during transport in 2 broiler hybrids with different growth rates, Ross 308, and Hubbard JA787, and to estimate the association between hybrid and mortality at different stages of the production cycle. The study sample consisted of 64,651,804 broilers from 4,228 flocks and 139 farms slaughtered from January 1st, 2015, to June 22nd, 2021. Analysis of on-farm mortality was restricted to broiler flocks slaughtered in the period July 2017 to July 2018 due to changes known to affect FWM. The effect of hybrid on mortality during 3 different production stages (first week mortality (**FWM**), mortality after the first week (**MAFW**), and mortality during transport (**DOA**)), was assessed using mixed effect negative binomial regression models. Descriptively, there were notable differences in mortality at all stages of the production cycle, with higher mean mortalities in Ross 308 (1.40% FWM, 3.05% MAFW, and 0.063% DOA) than in Hubbard JA787 (0.76% FWM, 1.49% MAFW, and 0.015% DOA). In the analysis, the largest estimated difference was found for dead on arrival under cold conditions, where mortality was almost 5 times higher in Ross 308 compared to Hubbard JA 787 (IRR: 4.70, 95% CI: 3.74–5.90). The estimated difference in FWM between hybrids was highest during autumn, with an estimated risk approximately 1.6 times higher in Ross than in Hubbard (IRR: 1.56, 95% CI: 1.30–1.86). For MAFW, the estimated risk was approximately 2 times higher in Ross than in Hubbard (IRR: 2.13 95% CI: 1.82–2.49). The findings in this study emphasize the need for more knowledge on causes of mortality in broilers including effects of genetic factors as basis for preventive measures.

## INTRODUCTION

Animal welfare is a multidimensional concept. According to the multicriteria evaluation model for animal welfare included in Welfare Quality, the 4 main welfare principles are: good feeding, good housing, good health and appropriate behavior (De Jong, 2011). Mortality is one measure suggested to reflect the principle of “Good health” in broiler chickens (de Jong et al., 2016). Accordingly, mortality, including culling, is an important and feasible outcome-based welfare measure during all stages of the production cycle (the Broiler Directive (CoEU, 2007)). Mortality is easy to recognize and record for the farmers, and widely used by veterinarians to evaluate broiler health and welfare (EFSA, 2010). Mortality in broiler production is most commonly reported as mortality risk, sometimes referred to as cumulative mortality, or simply mortality (Caffrey et al., 2017; Baxter et al., 2021; de Jong et al., 2022), defined as the number of dead and culled broilers divided by the number of broilers at risk at the start of the risk period (for on-farm mortality, all broilers placed in the house).

Throughout the production period on farm, various factors, including housing conditions, biosecurity routines, climatic control, season, stocking density, nutrition, growth rate, genetic susceptibility, and general management routines have been shown to affect mortality (Heier et al., 2002; de Jong and van Riel, 2020; Van Limbergen et al., 2020). During the first week, the risk factors for mortality further include breeder hen age and hatchery routines (Yassin et al., 2009; Yerpes et al., 2020), thus differing from the later production period (Van Limbergen et al., 2020). According to reports based on information from commercial production systems across Europe, mean first week mortality (**FWM**) ranges from 0.59 to 2.1% between studies (Yassin et al., 2009; Rayner et al., 2020; Van Limbergen et al., 2020; Yerpes et al., 2020; Baxter et al., 2021). Furthermore, recent field and experimental studies from production systems in Europe and Canada, report mean total on-farm mortalities during the production period ranging from 1.5 to 6.2% between studies (Rayner et al., 2020; Van Limbergen et al., 2020; Baxter et al., 2021; Torrey et al., 2021; de Jong et al., 2022). For the Norwegian broiler industry, the mean national on-farm mortality risk is annually reported at population level (2.64% in 2021; Animalia, 2022b). According to Norwegian legislation (MAF, 2001) all farmers are obliged to keep records of daily mortality; however, this information is not available for research purposes.

Mortality during transport to, and during lairage at the abattoir is termed dead on arrival (**DOA**) and is recorded separately from the mortality on farm. Each abattoir is required to record the DOA numbers according to European legislations (CoEU, 2007). Important risk factors described for DOA are the duration of transport (Vecerek et al., 2016), outdoor temperature (Vecerek et al., 2016), health status of the flock (Chauvin et al., 2011; Kittelsen et al., 2017), and stress or injuries related to catching (Chauvin et al., 2011; Kittelsen et al., 2017). In Norway, the national mean DOA number for all broilers transported to the abattoir was 0.06% in 2021 (Animalia, 2022b), whereas studies from the UK (Rayner et al., 2020; Baxter et al., 2021), report DOA of 0.04 and 0.1%. Furthermore, studies from Canada (Caffrey et al., 2017) and Brazil (Dos Santos et al., 2020) report DOA of between 0 to 19.4% and 0.11 to 0.22%, respectively.

In addition to numerous external factors, the health and robustness of the chickens is also influenced by genetics (EFSA, 2010). Indeed, recent studies under experimental conditions indicated that genetic selection for fast growth is an important risk factor for mortality in broiler chickens (Havenstein et al., 2003; Dixon, 2020). In addition, previous studies found associations between fast growth and adaptive immune system function (Cheema et al., 2003), metabolic function (Lubritz et al., 1995), lameness (Kestin et al., 2001), and feather condition (Ghayas et al., 2021). Studies comparing mortality between hybrids of different growth rates are scarce; however, 2 observational studies reported lower mortality during the first week and during the entire production period for slower growing broilers (Rayner et al., 2020; Baxter et al., 2021). They also reported fewer postmortem inspection rejections and overall lower gait scores, indicating beneficial effects of slower growth rates on several aspects relevant to animal health and welfare (Rayner et al., 2020; Baxter et al., 2021). In a recent study from Norway, including the same broiler cohorts as the present study, there were notable differences in the condemnation causes at the abattoir between hybrids with different growth rates (Ross 308 and Hubbard JA787) (Forseth et al., 2023). Although the use of slower growing broiler hybrids is increasing in developed countries (Davies, 2019; CiWF, 2020; Philippe, 2022), knowledge of on-farm mortality risk and of the association between growth rate and mortality at different stages during the production period and during transport under commercial production conditions is still limited.

The aim of this study was to describe the mortality risk in 2 hybrids with different growth rates (Ross 308 and Hubbard JA787) in commercial Norwegian broiler production, and to estimate the association between hybrid and mortality. Mortality risk was measured as 1) first week mortality (**FWM**), 2) mortality from the second week until slaughter (mortality after first week; **MAFW**), 3) total on-farm mortality, and 4) mortality during transport to slaughter (dead on arrival; DOA). A subsidiary aim was to explore seasonal differences in on-farm mortality and assess the effect of temperature on mortality during transport.

## MATERIALS AND METHODS

### Rearing and Transport Procedures

Broilers included in this study were housed according to Norwegian legislation standards (MAF, 2001) and the Norwegian broiler industry's Animal welfare program (Animalia, 2013). The broilers were kept in closed barns with automated ventilation and concrete flooring covered with bedding (mostly wood shavings). Feed was provided ad libitum in feeding pans with automatic filling, and water was provided by water lines with nipples and drip cups or cup drinkers. We did not have detailed information on nutrition and feed mills in the available data. Provided certain criteria are fulfilled, Norwegian legislation allows a maximum stocking density of 36 kg/m^2^ (MAF, 2001). In our study, median stocking density was 33.8 for Hubbard and 32.8 for Ross. For biosecurity reasons, the production was mainly an all-in-all-out, where all broilers from 1 house were delivered to slaughter in 1 slaughter batch (i.e., at the same time). However, for 24 of the 4,228 flocks in the dataset there was a risk of the stocking density exceeding the legal limit at the end of the cycle. For these flocks, part of the flock was slaughtered as 1 slaughter batch followed by the remaining part a few days later, often referred to as thinning. Before slaughter, all flocks were caught manually on farm and loaded in Marel Stork 4l4 transport crates (Marel Boxmeer, Handelstraat 3, 5831 AV Boxmeer). The crates were loaded on an enclosed truck with either negative pressure ventilation or positive pressure ventilation with heating and transported to the abattoir. We did not have information about transport duration. On arrival, each crate was checked to control the condition of the broilers, and all dead broilers were recorded and removed. All broilers in the present study were transported to, and slaughtered at, the same abattoir, for which the arrival area was closed with automated ventilation.

### Study Population

The source population of this retrospective observational study consisted of broiler cohorts from private farms contracted to the broiler company Norsk Kylling AS (**NK**). The study population consisted of flocks of the hybrids Ross 308 and Hubbard JA787 slaughtered in the period between January 1st, 2015, to June 22nd, 2021. The study unit was flock, that is, broilers housed together at the same farm. A farm denoted a site with 1 to 4 houses owned by 1 farmer. All farms were located in 2 counties in mid-Norway: Trøndelag and Innlandet (a map is included in the supplementary material Figure S1 online). Owner consent was obtained by signature on digital forms. Nonresponders were contacted by either email, SMS or by phone, followed by consent on SMS. During the study period, the broiler company made a transition from a fast growing to a slower growing hybrid over a period of 15 mo, starting in July 2017, and completed in October 2018. Consequently, both hybrids were represented in the population during this transition period. Prior to July 2017 all production units housed Ross only, and from the end of October 2018 onward, all holdings housed Hubbard.

### Data Sources, Variables, and Data Processing

Flock information and abattoir records of all flocks were retrieved from the broiler company's database “Production control”.

The database contained information from several sources: The number of broilers placed in each house was reported by the hatchery, while the number of broilers that died during the first 7 d of life (FWM) was reported by the farmer and recorded in the database. The number of broilers delivered to slaughter was the sum of the number of broilers approved for human consumption, registered automatically by an in-line counter, recordings of DOA from the arrival area and condemnations recorded by the meat inspectors. Broilers were weighed automatically after evisceration, and the average carcass weight was recorded in the database. Mean live weight per flock and stocking density were also available from the Production control database. The mean live weight is a variable estimated from dividing carcass weight by a conversion coefficient (0.68 for Hubbard, 0.678–0.681 for Ross depending on carcass weight) (Animalia, 2022a), while stocking density (kg/m^2^) is calculated by dividing total mean estimated liveweight (at slaughter day) by the area of the house (Stamp Dawkins et al., 2004).

The available count variables used in calculations of mortality were: The number of broilers placed on farm, the number of broilers dead within the first week after placement, the number of broilers delivered to slaughter and the number of broilers dead on arrival at the abattoir. From this we made 4 mortality variables: 1) first week mortality (FWM), 2) mortality from the second week until slaughter (MAFW), 3) total on-farm mortality from date of placement to slaughter, and 4) mortality during transport or lairage (DOA). The total number of broilers that died on farm (total on-farm mortality) was calculated by subtracting the number of broilers delivered to slaughter from the number of broilers placed on farm. On-farm mortality after first week (MAFW) was calculated by subtracting the number of broilers dead on farm within the first 7 d from the total number of broilers dead on farm. FWM and MAFW included birds found dead and culled birds, as they were recorded together in the available data. The DOA-variable was directly available in the database and did not require any calculations. The mortality variables FWM, MAFW and DOA were all calculated as counts per flock to be used for further calculations of mortality risks.

The age at slaughter was calculated by subtracting the date of placement from the slaughter date. To account for seasonal variations, a variable for season, based on date of slaughter, was categorized as follows: winter: December to February, spring: March to May, summer: June to August, and autumn: September to November. Outdoor temperatures on the slaughter date were retrieved from Norwegian Centre for Climate Services (NCCS). As the broilers were transported during the night, we chose to use the minimum daily air temperature. This variable was characterized into 4 temperature categories of approximately equal intervals. All variables and the hierarchical structure in the dataset are described in Table 1.

**Table 1:** Descriptive overview of flock information, variables and the hierarchy of the final dataset consisting of 4,228 flocks from 139 Norwegian poultry farms housed during the time period 2015 to 2021.

Variable	Type of variable	Level in hierarchy	Comments/judgment
Farm ID	Nominal	Highest level	Unique identifier for each of the 139 farms included. Highest level in the hierarchy
House	Nominal		Every farm has 1 to 4 broiler houses, median value was 1
Flock ID	Nominal	Lowest level	Unique identifier of each flock, broilers from the same farm, housed in the same house. Lowest level in the hierarchy.
Hybrid	Nominal		Two cross-bred broiler hybrids developed for meat production
Date placed	Continuous		Date for placement of broilers in the house
Slaughter date	Continuous		Date of arrival at the abattoir
Number placed	Discrete		The number of broilers placed in the broiler house
Number delivered to slaughter	Discrete		The number of broilers shipped to the abattoir
Age	Ordinal		Age of broilers at slaughter. Calculated by subtracting the date of placement from the slaughter date.
Weight	Continuous		Carcass weight at slaughter after plucking and evisceration
Density	Continuous		Estimated stocking density in the broiler house at the end of the production period.
Season	Nominal		Variable for seasons of the year: winter (December–February), spring: (March–May), summer: (June–August), autumn: (September–November)
Temperature	Continuous		Variables for minimum air temperatures per day (24 h) on slaughter date. 0 (−23.6°C to −12°C), 1 (−12°C to −2°C), 2 (−2°C to 7°C) and 3 (7°C–16.6°C).
First week mortality (FWM)	Discrete		The number of broilers dead on farm during the first 7 d after placement. Recorded by the farmer.
Restmortality	Discrete		The number of broilers dead on farm from d 8 to slaughter date. Calculated by subtracting first week mortality from total mortality.
Total on-farm mortality	Discrete		The number of broilers dead on farm from date of placement to shipment for slaughter. Calculated by subtracting number delivered to slaughter from number placed.
Dead on arrival (DOA)	Discrete		The number of broilers dead during transport from farm to abattoir or during lairage at the abattoir. Recorded at the abattoir.

Data were stored in Microsoft Excel 365, while StataSE 17.0 (Stata, 2021) was used for data management and statistical analysis.

### Measures of Mortality

We use the term mortality risk to denote the cumulative mortality (%) during the different periods of production: 1) first week mortality, 2) mortality from the second week until slaughter 3) total on-farm mortality, and 4) mortality during transport or lairage. The raw mortality risk was calculated for each of 3 different groups: the entire study population, each hybrid separately, and finally for each flock. At the population level, the mortality risks were calculated as: 1) total FWM risk; the total number of birds that died during the first week divided by the total number placed throughout the study period. 2) Total MAFW risk; the total number of birds that died from d 8 until shipping to slaughter, divided by the number of broilers left in all houses after the first week (total number placed minus total FWM). 3) Total on-farm mortality risk, calculated by dividing all broilers that died during the period from placement to shipping by the total number of broilers placed during the study period. 4) Total DOA risk was calculated by dividing total DOA by the total number of birds delivered to slaughter. All risks were then multiplied by 100 to achieve percentages. Following the same approach, we calculated the overall mortality risk also for each hybrid separately.

All measures of mortality (FWM risk, MAFW risk, Total on-farm mortality risk and DOA risk), was also calculated at flock level, analogous to the approach described above. As for the population level risks, all flock level mortality risks are presented as percentages. The distribution of FWM and MAFW at flock level was visualized across the study period, and for each of the 4 seasons (winter, spring, summer, and autumn) by creating box plots. In the box plots for on-farm mortality, 5 and 4 extreme values (FWM risk >10 and MAFW risk >15), were omitted for FWM and MAFW, respectively, to ease the readability of the figures. Similarly, box plots were generated to describe the DOA mortality risk per flock across the study period, and in each of the 4 temperature categories (0 (−23.6°C to −12°C), 1 (−12°C to −2°C), 2 (−2°C to 7°C), and 3 (7°C–16.6°C)).

### Inclusion Criteria and Data Cleaning

The inclusion criteria were flocks consisting of broilers of the hybrids Ross 308 (Ross) or Hubbard JA787 (Hubbard), slaughtered during the study period from January 2015 to June 2021.

During data cleaning, errors, unlikely and missing values were checked against food chain information and replaced with correct values if these were possible to obtain. If correct information was unavailable, the flock was excluded from the study. Extreme mortalities caused by known accidents such as, for example, ventilation failure, and traffic accidents during transport were excluded from the study. Flocks of hybrids apart from Ross 308 and Hubbard JA787 were also excluded.

**Figure 1:**
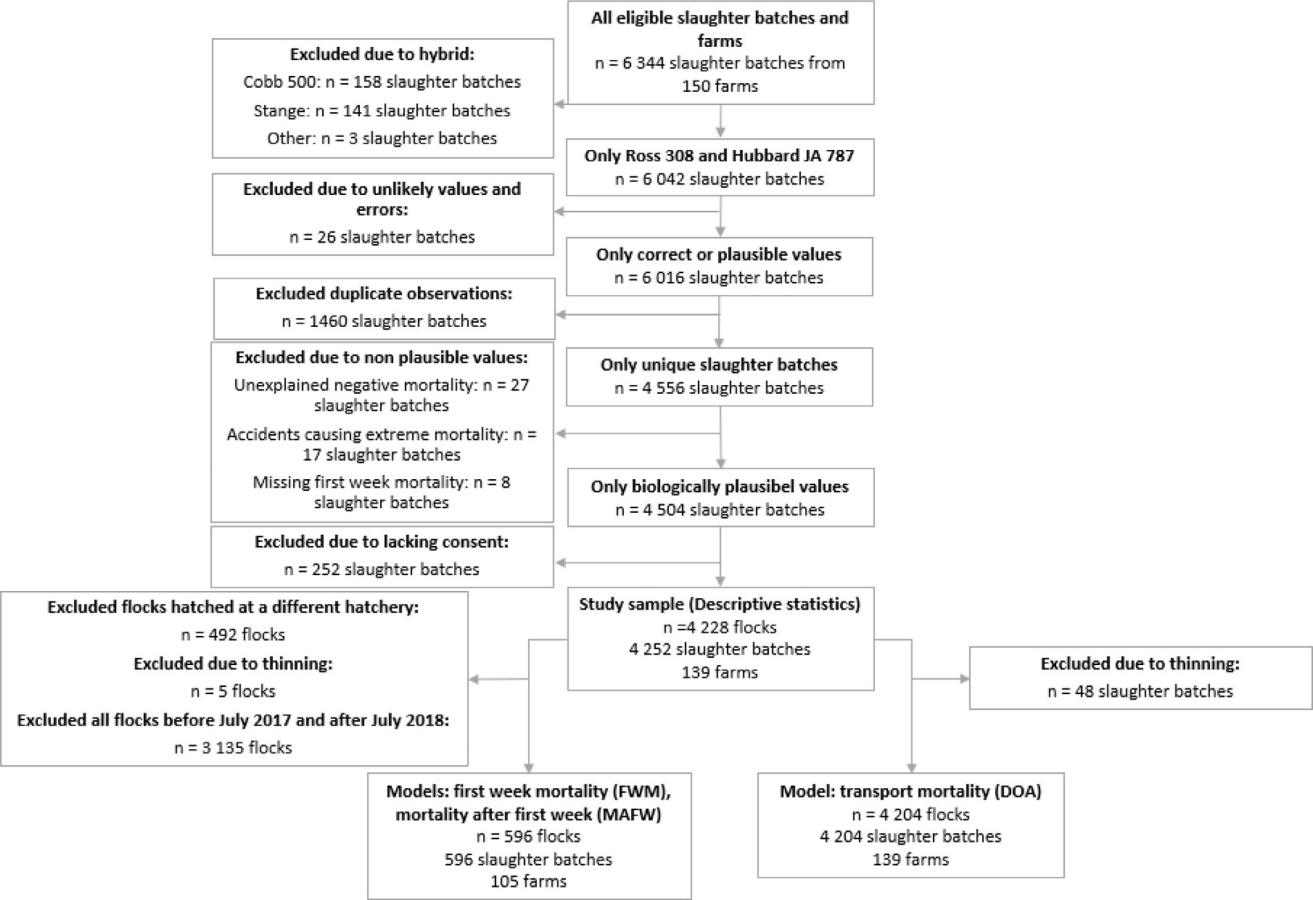
Flowchart describing all eligible flocks, exclusions, and the final study samples used in all analyses.

Descriptive statistics were calculated for a study sample of 4,228 flocks after data cleaning (Figure 1). For the statistical analysis, some additional exclusions were made: For the statistical analysis of on-farm mortality, 492 Ross flocks hatched at a different hatchery than the rest were excluded to avoid confounding. Also, due to changes in hatchery routines in 2016, known to affect first week mortality numbers, the study population for the statistical analysis of on-farm mortality was restricted to broiler flocks slaughtered in the period July 2017 to July 2018. In this period there was an overlap between the 2 hybrids, thus reducing the risk of confounding caused by changes in routines for only one of the hybrids. Thinned flocks were excluded from the analyses, as it concerned only a very limited number of flocks and was unevenly distributed between the 2 hybrids. A description of the data cleaning process, showing all eligible, excluded, and analyzed flocks can be found in Figure 1.

### Descriptive Statistics

Descriptive statistics of mortality are presented for the entire study population, for each hybrid separately and for each flock, using median, range and interquartile range. For flock level measures of mortality, we assessed correlations between FWM risk and MAFW risk, between FWM risk and DOA risk and between MAFW risk and DOA risk by calculating Spearman rank correlation. The correlations were visualized using scatterplots and Lowess smoothed curves. As for the box plots, extreme values (FWM risk >10 and MAFW risk >15), were omitted for FWM (*n* = 5) and MAFW (*n* = 4), respectively, to ease the readability of the figures.

### Statistical Analysis Methodology

***The Association Between Hybrid and Mortality.*** As we had aggregated data for mortality, recorded as counts per flock, flock was the unit of all statistical analyses.

To assess the effect of hybrid on the different categories of mortality, we used (flock-counts of) FWM, MAFW and DOA as outcome variables. The effect of hybrid was assessed using mixed effects negative binomial models for each of the 3 outcome variables separately. Negative binomial models were considered more appropriate than Poisson models due to overdispersion, that is, a conditional variance of the outcome variable larger than the conditional mean, tested by likelihood ratio testing of the overdispersion parameter alpha equal to zero. The number of broilers placed in the house was set as the exposure variable in the 2 models of on-farm mortality (FWM and MAFW). For DOA, the number of broilers submitted to slaughter was set as the exposure variable. To account for the hierarchical structure of the data, farm was included as random effect for all 3 models.

**Figure 2:**
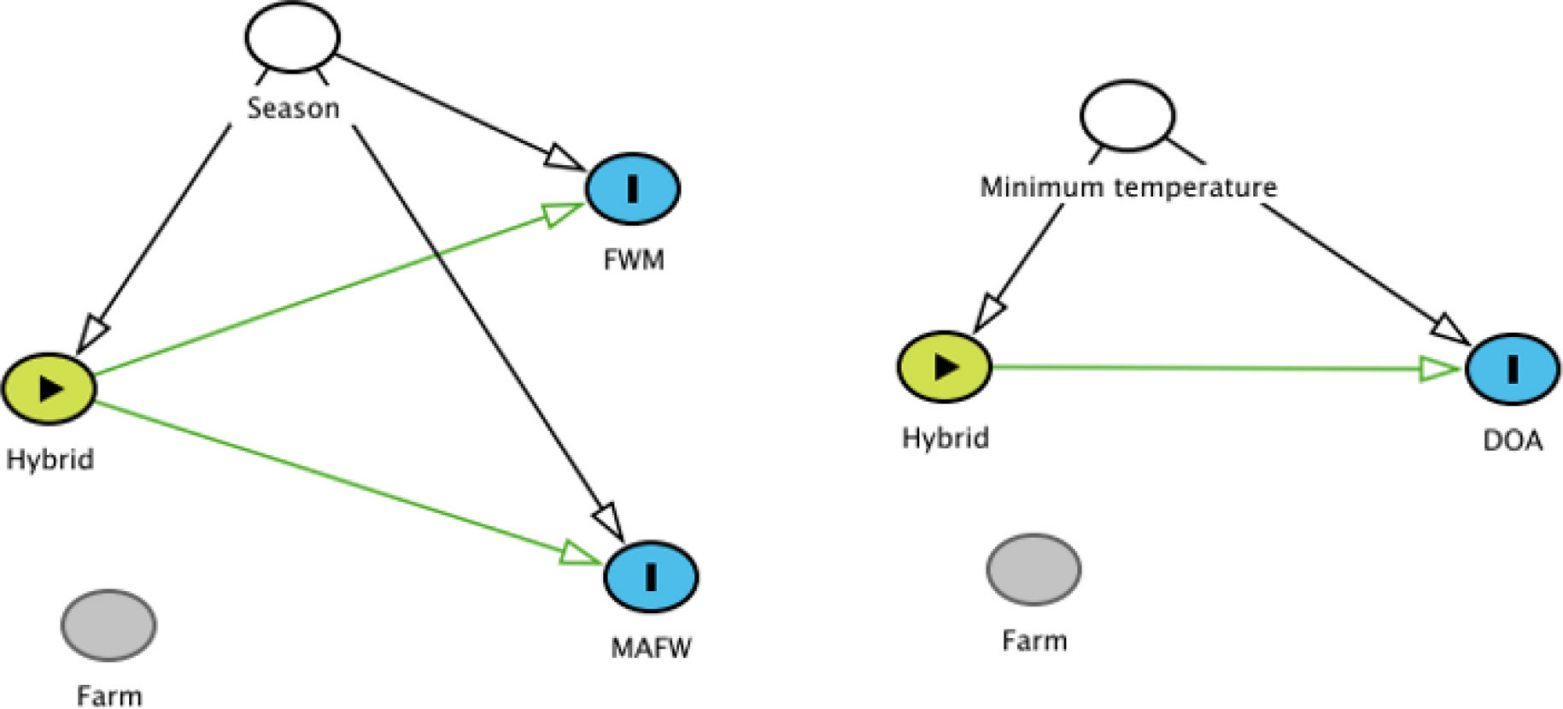
Causal diagrams for the final models of mortality during the first week of the production period (FWM), mortality after first week (MAFW) (broilers dead from d 8 to slaughter) and mortality for broilers dead on arrival (DOA).

Prior to analysis, potential causal pathways and links between the exposures and outcome variable were described and illustrated using directed acyclic graphs (**DAGs**). The DAGs were based on literature reviews and discussion among the authors and created using the software Daggity (Textor et al., 2016). The final DAGs are shown in Figure 2. Variables that were dependent on farm of origin, for example ventilation and nutrition, were considered “farm effects” and were not included in the DAGs. In all regression models, broiler hybrid was the exposure variable of primary interest, and inclusion of confounders was based on the directed acyclic graphs. Additionally, the importance of these confounders was assessed by monitoring the change in the coefficient for hybrid when the confounder was included in the model and when it was not included. Season was tested as potential confounders in the on-farm models (FWM and MAFW). For the DOA-model we offered temperature as a potential confounder. Slaughter age and carcass weight were both highly collinear with hybrid and were therefore tested for each hybrid separately; however, no statistically significant effect was found (*P* > 0.05, results not shown). Stocking density was measured at the end of the production period and was not considered for inclusion as the causal pathway between mortality and animal density is likely reversed (i.e., high mortality causes lower density).

Biologically plausible interactions were tested for all 3 models by adding the interaction between each term and hybrid to the model. Interaction terms were only kept in the model if *P* < 0.01, for the sake of model parsimony. The final model for FWM and MAFW included hybrid, season and their interaction as fixed effects and farm as a random effect to account for clustering of flocks from the same farm. The final model for DOA included hybrid, minimum temperature, and their interaction as fixed effects and farm as a random effect. Regression diagnostics were performed for each model by computing standardized Pearson and deviance residuals to determine whether the final models adequately represented the data. Normality plots were used to assess the distribution of residuals and the residuals were plotted against predicted values. Inspection of residual plots revealed no major shortcomings of the models. The effect of high mortality flocks on model estimates was evaluated by removing observations above the 90th percentile and rerunning the model without these observations. The model was also rerun omitting all observations with standardized Pearson residual <−3 or >3. The proportion of the unexplained variation in the outcome at the 2 levels of the hierarchy, was assessed by calculating variance proportion components following the method described by Leckie et al. (2020).

For the DOA model, the subset of the data truncated to the time period where there was an overlap between the 2 hybrids (July 2017–July 2018) was used in a sensitivity analysis. The model was re-run using this dataset and compared to the primary analysis to evaluate the robustness of the obtained estimates.

## RESULTS

### Descriptive Statistics

After data cleaning, the final study sample for the on-farm mortality models consisted of a total number of 64,651,804 broilers, 34,768,415 Hubbard and 29,883,389 Ross. Of these, 2,402 were Hubbard flocks, and 1,826 were Ross flocks. The broilers came from 4,228 flocks placed in 139 farms during the 6-yr study period (2015–2021). The median age at slaughter was 46 d for Hubbard and 33 d for Ross, and median weight at slaughter was 1,654 g for Hubbard and 1,356 for Ross (Table 2). The overall median stocking density for all flocks was 33.5 kg/m^2^. For Ross, the median stocking density was 32.8 kg/m^2^, and for Hubbard 33.78 kg/m^2^.

**Table 2:** Descriptive statistics of 4,228 broiler flocks slaughtered during the study period (January 2015–June 2021) for Hubbard JA787 and Ross 308.

		Flock				
Item	Total no.	Median	Min	Max	Q1	Q3
Hybrid	Hubbard JA787					
No placed on farm	34,768,415	14,000	4,300	38,000	12,200	15,500
No delivered to slaughter	33,990,075	13,683	4,084	37,141	11,849	15,224
Carcass weight at slaughter (g)		1,654	1,279	1,968	1,589	1,707
Age at slaughter (d)		46	39	50	46	47
Hybrid	Ross 308					
No placed on farm	29,883,389	16,400	5,250	35,000	14,300	18,200
No delivered to slaughter	28,565,384	15,649	4,904	29,841	13,704	17,437
Carcass weight at slaughter (g)		1,356	775	1,737	1,267	1,450
Age at slaughter (d)		33	28	37	32	34

***On-Farm Mortality.*** For all broilers placed on farm (regardless of hybrid), 2,096,345/64,651,804 (3.24%) broilers died on farm during the entire production period. During the first 7 d after placement 682,731/64,651,804 (1.06%) broilers died, and 1,413,614/64,651,804 (2.21%) broilers died on farm during the period from d 8 to slaughter. When differentiating between the 2 hybrids, the total on-farm mortality risk was higher for Ross (4.41%) than for Hubbard (2.24%) (Table 3).

**Table 3:** The total on-farm mortality (*n* = 64,651,804 placed broilers, *n* = 139 farms), and mortality during transport (DOA) (*n* = 62,555,459 broilers delivered to slaughter, *n* = 139 farms), for Hubbard JA787 and Ross 308 during the study period from January 2015 to June 2021.

	Hubbard JA787		Ross 308	
Measures of mortality	Number of broilers	Percent	Number of broilers	Percent
First week mortality (FWM)	264,408	0.76%	418,323	1.40%
Mortality after first week (MAFW)	513,932	1.49%	899,682	3.05%
Total mortality (d 0–slaughter)	778,340	2.24%	1,318,005	4.41%
Transport mortality (DOA)	5,145	0.015%	17,888	0.063%

**Figure 3:**
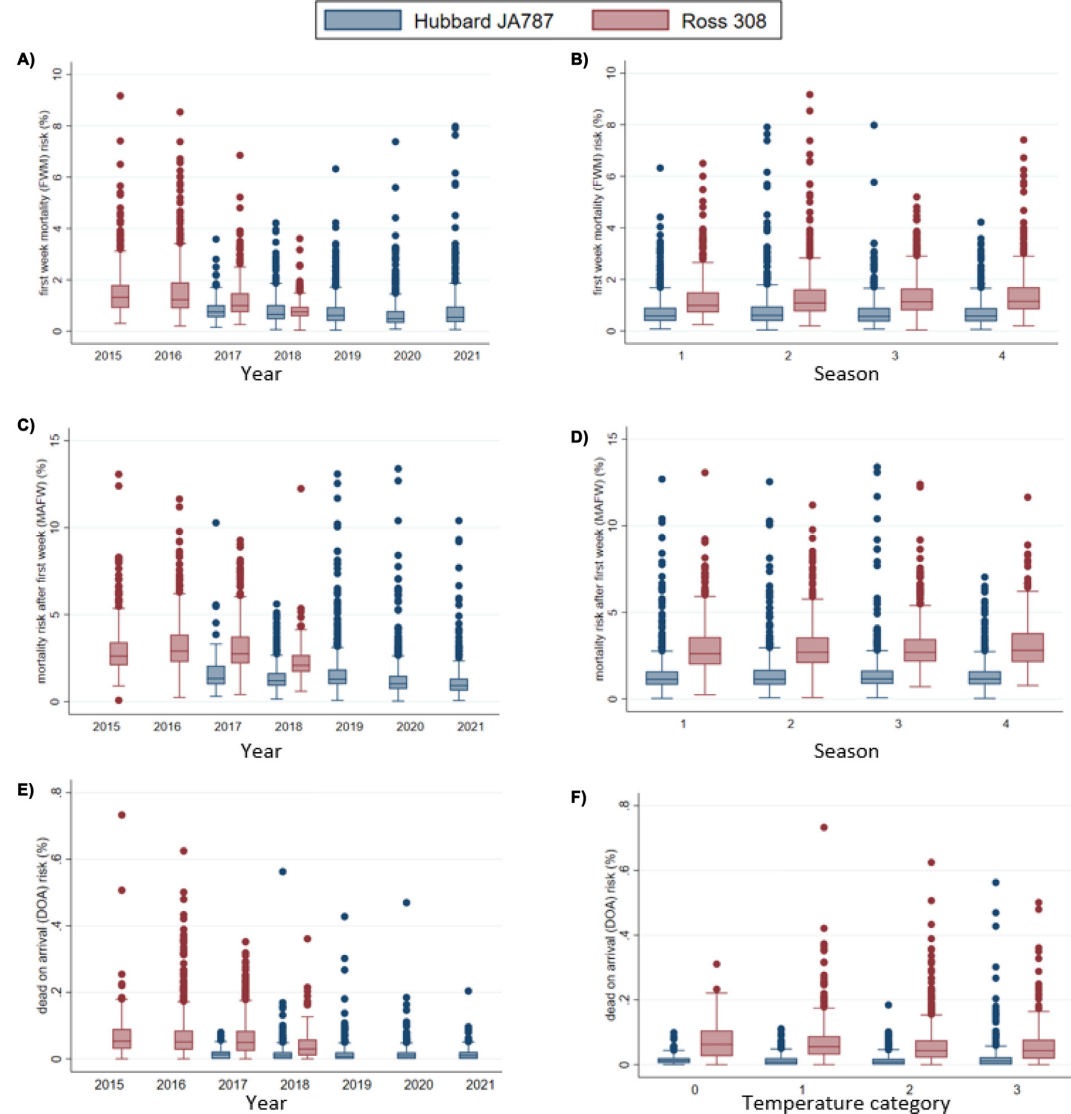
Boxplots showing flock level raw mortality risks during the production periods: first week of the production period (FWM), in panel (A) across the entire study period (2015–2021) and (B) for the 4 seasons (1 = winter, 2 = spring, 3 = summer, and 4 = autumn), mortality risk after first week (MAFW) (broilers dead from d 8 to slaughter) in panel (C) across the entire study period and (D) for the 4 seasons, and broilers dead on arrival (DOA) at the abattoir in panel (E) across the entire study period and (F) for each of 4 outside temperature categories (0 (−23.6°C to −12°C), 1 (−12°C to −2°C), 2 (−2°C to 7°C) and 3 (7°C–16.6°C)) for the 2 hybrids Hubbard JA787 (*n* = 2,401 flocks for FWM and MAFW and *n* = 2,395 flocks for DOA) and Ross 308 (*n* = 1,822 flocks for FWM and MAFW and *n* = 1,809 flocks for DOA)).

At flock level, the on-farm mortality risks were higher for Ross than for Hubbard both during the first week and the rest of the production period (Table 4). The flock level FWM was notably higher in Ross than in Hubbard during most of the study period; apart from 2018 where the difference was smaller (Figure 3A)). When assessing seasonal variations in FWM, Ross flocks had an overall higher mortality for all 4 seasons (Figure 3B)). Also, for the MAFW, the overall proportion of birds that died was higher for Ross, compared to Hubbard (Table 4), and at flock level, the distribution of MAFW risk was higher in Ross flocks compared to Hubbard flocks for all the included years (Figure 3C)). Regarding seasonal variations, the pattern was similar to FWM, with a higher mortality in Ross flocks for all 4 seasons (Figure 3D)).

**Table 4:** Flock level on-farm mortality risk and transport mortality (DOA) risk for Hubbard JA787 and Ross 308 (*n* = 2,402 and 1,826 flocks, respectively), during the study period from January 2015 to June 2021.

	Hubbard JA787			Ross 308		
		IQR			IQR	
Mortality	Median	Min	Max	Median	Min	Max
First week mortality (FWM)	0.59%	0.38%	0.91%	1.08%	0.79%	1.60%
Mortality after first week (MAFW)	1.14%	0.83%	1.63%	2.71%	2.09%	3.58%
Total mortality	1.84%	1.36%	2.57%	3.84%	3.01%	5.08%
Transport mortality (DOA)	0.009%	0%	0.020%	0.047%	0.024%	0.082%

***Mortality During Transport and Lairage (DOA).*** The final study sample included in calculations of DOA consisted of a total number of 62,555,459 broilers, 33,990,075 Hubbard and 28,565,384 Ross delivered to slaughter throughout the 5-yr study period (2015–2021). These broilers came from 4,228 flocks and 139 farms. Per hybrid, the study sample included 2,402 Hubbard flocks and 1,826 Ross flocks. For all broilers delivered to slaughter, regardless of hybrid, 23,033/62,555,459 (0.037%) broilers died during transport and lairage. The overall DOA risk was notably higher for Ross (0.063%) than for Hubbard (0.015%) (Table 3). At flock level, the median mortality risk was higher for Ross than for Hubbard (Table 4). The flock distribution of DOA was higher in Ross than in Hubbard for all the included years (Figure 3E)). Furthermore, the DOA risk was higher for Ross than for Hubbard across all temperature categories (Figure 3F)). In total, 807/4,228 flocks had zero DOA, with a notable difference between hybrids; 704/2,402 (29%) Hubbard flocks and 103/1,826 (6%) Ross flocks had no birds DOA.

**Figure 4:**
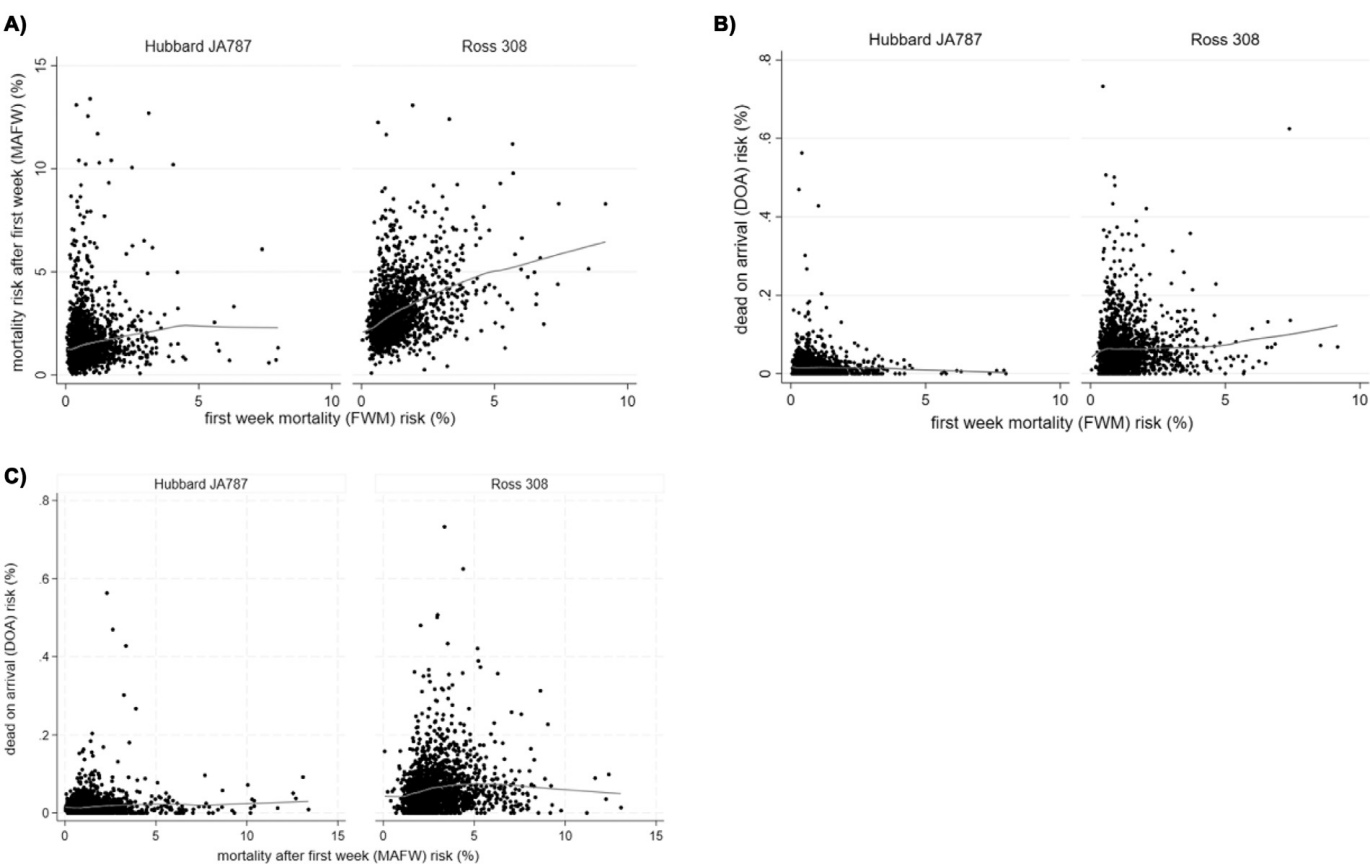
Lowess smoother curves describing the correlation between (A) first week mortality (FWM) risk and mortality risk after first week (MAFW) for the 2 hybrids Hubbard JA787 (*n* = 2,401 flocks) and Ross 308 (*n* = 1,822 flocks), (B) between first week mortality (FWM) risk and transport mortality (DOA) risk for each of the 2 hybrids, Hubbard JA 787 (*n* = 2,394 flocks) and Ross 308 (*n* = 1,805 flocks), and (C) between mortality risk after first week (MAFW) and transport mortality (DOA) risk for each of the 2 hybrids, Hubbard JA 787 (*n* = 2,392 flocks) and Ross 308 (*n* = 1,803 flocks).

***Correlations Between FWM and MAFW, Between FWM and DOA, and Between MAFW and DOA.*** Correlations were assessed visually, and as can be seen in Figure 4, the MAFW increased with increasing FWM in both Ross and Hubbard flocks. However, the correlation between FWM and MAFW was moderate in Ross (Spearman rank correlation, *ρ* = 0.4176) and low in Hubbard (Spearman rank correlation, *ρ* = 0.2067) (Figure 4A)). We found no correlation between FWM and DOA (*ρ* = 0.0536 for Ross and *ρ* = 0.0213 for Hubbard) or between MAFW and DOA (*ρ* = 0.1563 for Ross and *ρ* = 0.0661 for Hubbard) (Figure 4B and C, respectively) for any of the hybrids.

### Statistical Analysis

**Figure 5:**
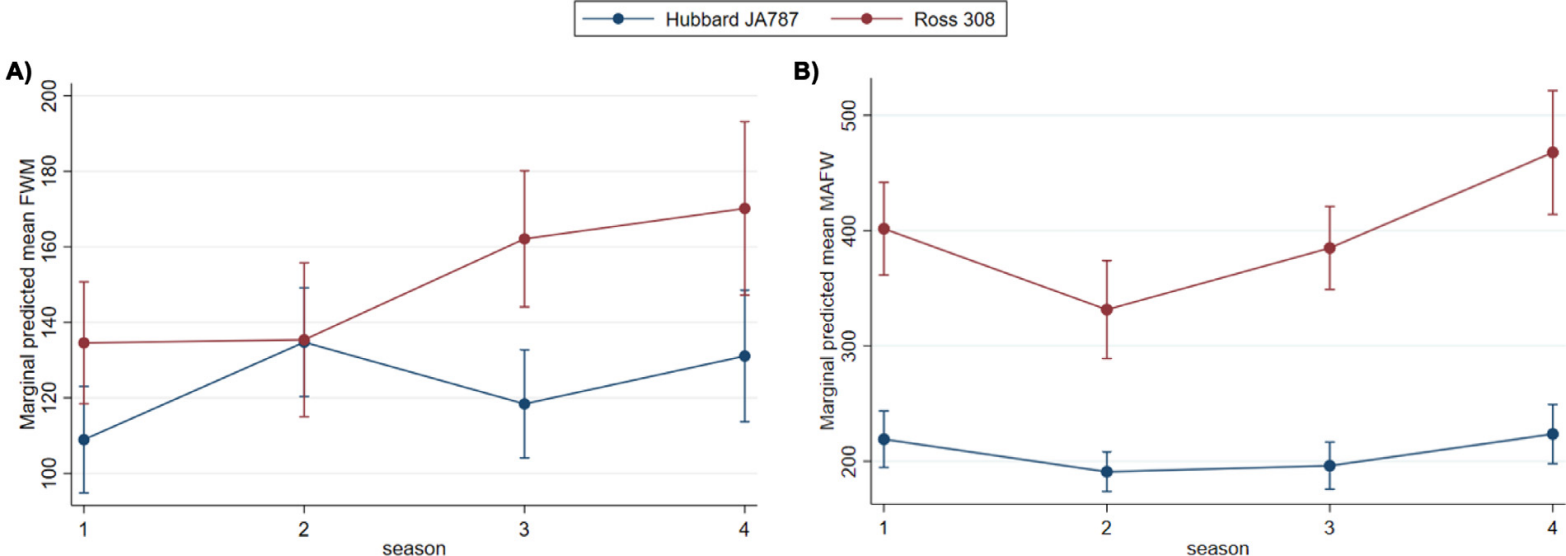
Marginal predicted mean mortalities in the 2 broiler hybrids Hubbard JA787 (*n* = 303 flocks) and Ross 308 (*n* = 293 flocks) across seasons (1 = winter: December, January and February(*n* = 151 flocks), 2 = spring: March, April and May (*n* = 160 flocks), 3 = summer: June, July and August (*n* = 167 flocks), 4 = autumn: September, October, and November (*n* = 123 flocks) in (A) first week mortality (FWM) and (B) mortality after first week (MAFW) (note the proportion of broilers of each hybrid varied throughout this year).

***The Association Between Hybrid and Mortality on Farm (FWM and MAFW).*** The results from the negative binomial regression models assessing the associations between hybrid and the on-farm mortality measures; FWM and MAFW are shown in Table 5. The model estimating the association between hybrid and FWM included an interaction term between hybrid and season. The largest estimated difference between hybrids was found during autumn, where the estimated risk was approximately 1.6 times higher in Ross than in Hubbard IRR (95% CI): 1.56 (1.31–1.86). For Ross the FWM was lowest during winter and spring, and higher in the summer and autumn. For Hubbard, the highest predicted FWM was found in the spring (Figure 5A)). Calculations of the proportion of unexplained variance showed that the mean variance proportion component (**VPC**) was lower at the farm level (VPC mean: 30.2% (Hubbard) and 30.5% (Ross)) compared to flock level (VPC mean: 69.8% (Hubbard) and 69.5% (Ross)), indicating that variation between flocks contributed more to the unexplained variation in the outcome compared to between farm variation.

**Table 5:** Results of the mixed effects negative binomial models for first week mortality (FWM) and mortality from d 8 to slaughter (MAFW). In both models a hybrid - season interaction term was included (winter: December, January and February, spring: March, April and May, summer: June, July and August, autumn: September, October, and November). The study sample consisted of 105 farms and 596 flocks of 2 different hybrids (Hubbard JA787 and Ross 308) slaughtered from July 2017 to July 2018.

Model	Variables	IRR	SE	*P* value
First week mortality (FWM)	Hybridxseason			
	Hubbard winter	Baseline		
	Hubbard spring	1.233	0.089	0.004
	Hubbard summer	1.082	0.084	0.312
	Hubbard autumn	1.199	0.097	0.025
	Ross winter	1.231	0.104	0.014
	Ross spring	1.239	0.120	0.027
	Ross summer	1.488	0.117	<0.001
	Ross autumn	1.557	0.140	<0.001
	Random effect variances			
	Farm	0.086	0.017	
				
Mortality after first week (MAFW)	Hybridxseason			
	Hubbard winter	Baseline		
	Hubbard spring	0.872	0.052	0.04
	Hubbard summer	0.893	0.064	0.115
	Hubbard autumn	1.017	0.076	0.821
	Ross winter	1.829	0.135	<0.001
	Ross spring	1.509	0.129	<0.001
	Ross summer	1.754	0.113	<0.001
	Ross autumn	2.130	0.169	<0.001
	Random effect variances			
	Farm	0.039	0.010

As for the FWM model, the model estimating the association between hybrid and MAFW included an interaction term between hybrid and season. Again, the largest estimated difference in MAFW risk was found during autumn, where the estimated risk was approximately 2 times higher in Ross than in Hubbard IRR (95% CI): 2.14 (1.82–2.49). For Ross, the lowest predicted MAFW was found during spring, and the highest during autumn, whereas for Hubbard, the seasonal differences were minor for MAFW (Figure 5B)). As for FWM, the proportion of unexplained variance in MAFW was quite low at the farm level (VPC mean: 19.0% (Hubbard) and 19.3% (Ross)) relative to the flock level (VPC mean: 81.0% (Hubbard) and 80.7% (Ross)), indicating that most of the unexplained variation was between flocks.

**Figure 6:**
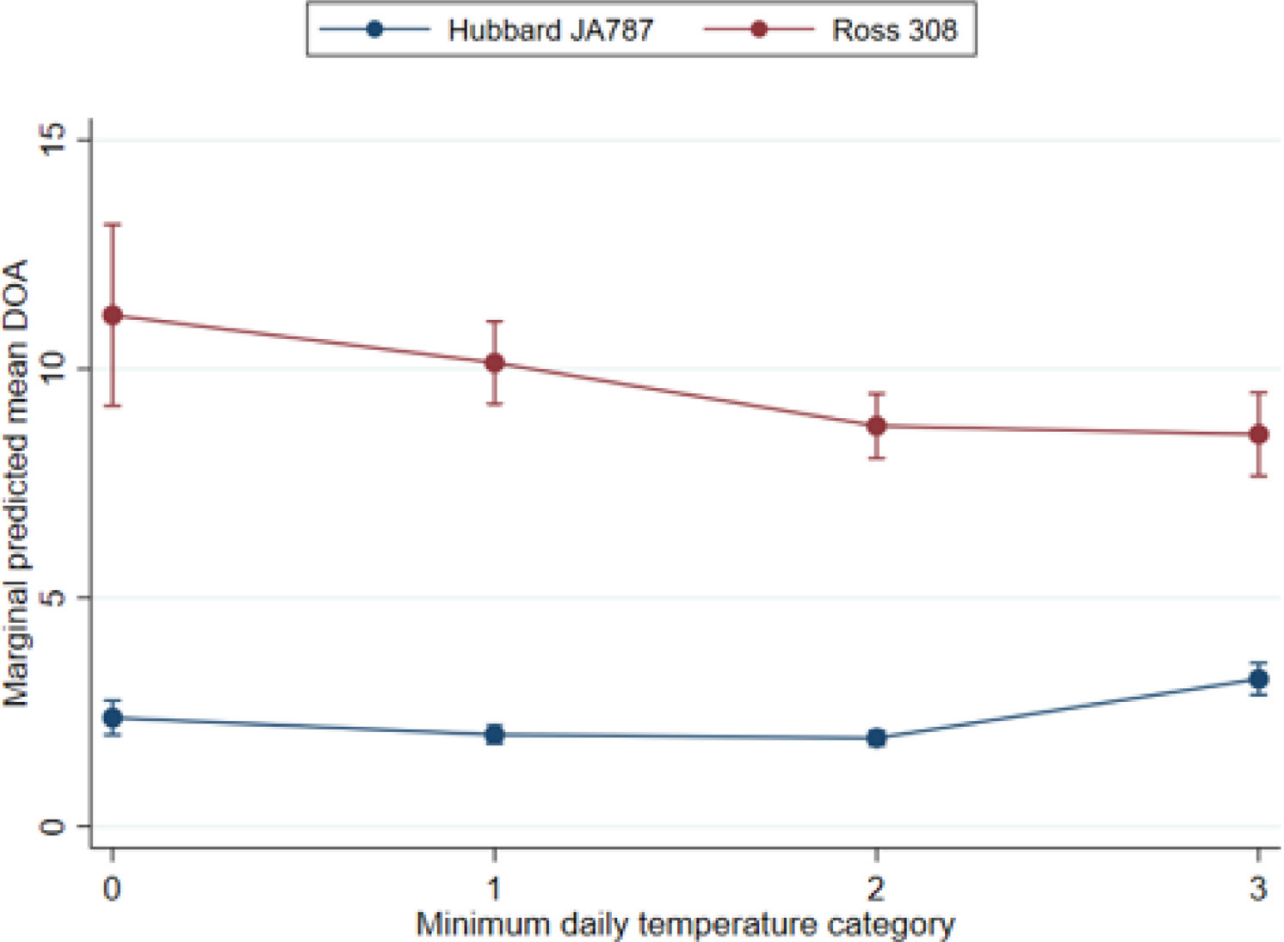
Marginal predicted mean mortalities in the 2 broiler hybrids Hubbard JA787 (*n* = 2,395 flocks) and Ross 308 (*n* = 1,809 flocks) across minimum daily temperatures (0 = (−23.6°C to −12°C), 1 = (−12°C to −2°C), 2 = (−2°C to 7°C) and 3 = (7°C–16.6°C)) during transport (DOA).

***The Association Between Hybrid and Mortality During Transport and Lairage (DOA).*** When comparing estimates for the different production periods, the strongest association between hybrid and mortality was found for DOA, where the estimated mortality risk was overall higher in the fast-growing Ross than in the slower growing Hubbard. There was a significant interaction term between minimum daily air temperature and hybrid, that is, the effect of hybrid was not the same for all temperatures. The largest estimated difference in DOA was under the coldest temperature conditions, for which the estimated risk was 4.7 times higher for Ross than for Hubbard (IRR: 4.70, 95% CI: 3.74–5.90) (Table 6). For Ross, the highest estimated DOA was found in the lowest temperature category with a decreasing trend toward warmer temperatures (Figure 6). For Hubbard, there were only minor differences between temperature categories; however, the predicted number of DOA was highest for the highest temperature category (Figure 6). Inspection of high mortality flocks (DOA > 90th percentile) showed that the majority of these were Ross flocks; of the 392 flocks, 369 were Ross and 23 were Hubbard. Due to the many high mortality Ross flocks, these observations influence the model estimates, and when omitted, the IRR is on average lowered by 36%. Omitting observations with Standardized Pearson residuals <−3 or >3 had negligible effect on the model estimates. The proportion of unexplained variance in DOA was low at the farm level (VPC mean: 6.1% (Hubbard) and 8.8% (Ross)) indicating that most of the unexplained variation was at the flock level (VPC mean: 93.9% (Hubbard) and 91.2% (Ross)). For the DOA model, the change in coefficients was negligible (change in coefficient <2%) in the transition period July 2017 to July 2018 compared to the entire study period, IRR (95% CI): 4.63 (2.94–7.30).

**Table 6:** Results of the mixed effects negative binomial models for transport mortality (DOA), including an interaction between hybrid and minimum temperature (0 = (−23.6°C to −12°C), 1 = (−12°C to −2°C), 2 = (−2°C to 7°C) and 3 = (7°C–16.6°C)). The study sample consisted of 139 farms and 4,204 flocks slaughtered from January 2015 to June 2021.

Model	Variables	IRR	SE	*P* value
Transport mortality (DOA)	Hybridxmintemp			
	Hubbard 0	Baseline		
	Hubbard 1	0.845	0.074	0.055
	Hubbard 2	0.813	0.068	0.014
	Hubbard 3	1.357	0.123	0.001
	Ross 0	4.703	0.545	<0.001
	Ross 1	4.264	0.365	<0.001
	Ross 2	3.683	0.308	<0.001
	Ross 3	3.606	0.327	<0.001
	Random effect variances			
	Farm	0.076	0.013

## DISCUSSION

To our knowledge, this is the first large scale study to describe mortality risk of, and to estimate associations between 2 broiler hybrids with 2 different growth rates (Ross 308 and Hubbard JA787) in commercial Norwegian broiler production. Briefly, we found that the mortality risk was higher in the fast-growing Ross throughout the production period, including mortality during the first week (FWM), from d 8 until shipping (MAFW), and during transport to slaughter (DOA). Statistical analyses showed higher estimated risks of mortality in Ross than in Hubbard in these production periods, and seasonal differences in on-farm mortality and effects of temperature on DOA were identified.

Total FWM was higher in Ross (1.40%) than in Hubbard (0.76%). The model estimating the association between hybrid and FWM showed largest estimated difference between hybrids during autumn, where the estimated mortality risk was approximately 1.6 times higher in Ross than in Hubbard. The findings of higher FWM in fast-growing broilers are in accordance with earlier studies (Rayner et al., 2020; Baxter et al., 2021). The underlying mechanisms behind a higher FWM in the faster growing hybrid are not known. Selective breeding for increased broiler performance could result in a negative impact on the immune system which in turn could increase disease susceptibility and risk of infectious diseases as suggested by Cheema et al. (2003). To the author's knowledge, there are no recent studies investigating and comparing the causes of FWM in different broiler hybrids; however, infectious diseases caused by pathogens like *E. coli* and culls of runts and lame broilers are known causes of FWM. A full risk factor analysis of FWM was not performed in the present study due to lack of information on important risk factors. However, in addition to genetic factors, others have reported that early chick development can be affected by age of breeder hen, egg weight, conditions during incubation and hatching, access to feed, nutritional value of the feed, age of house, floor quality, climatic conditions, and farm management (Vieira and Moran, 1999; Heier et al., 2002; Van Limbergen et al., 2020; Yerpes et al., 2020). We report a higher estimated FWM in Ross than in Hubbard in winter, summer, and autumn, while in spring, the results were similar between the hybrids. Seasonal variations in FWM have been described in earlier studies (Yassin et al., 2009; Yerpes et al., 2020), however the causal factors behind seasonal differences are not fully understood. It should be noted that due to changes in the hatchery's routines during the study period in the present study, the analysis for FWM was restricted to only 1 yr. In addition, the proportion of broilers of each hybrid varied throughout this year. Altogether, this means that conclusions regarding the seasonal effects on FWM cannot be made from the current study and longitudinal studies across several years would be preferable to investigate this further.

We found that MAFW was higher in the fast-growing Ross (3.05%) than in the slower growing Hubbard (1.49%). As for the FWM model, the largest estimated difference between hybrid and MAFW was found during autumn, with an estimated risk approximately 2 times higher in Ross than in Hubbard. A full risk factor analysis was not performed in our study. However, a recent study using the same study population as the current, reported differences in condemnation causes between the 2 hybrids (Forseth et al., 2023). Several chronic health conditions, like ascites, growth retardation, and hepatitis, were more common in the fast-growing Ross than in Hubbard (Forseth et al., 2023). It thus seems likely that the differences in mortality found in this study are related to the overall flock health. Other reported risk factors for on-farm mortality include housing conditions, biosecurity routines, season, stocking density, nutrition, growth rate, and general management routines (Heier et al., 2002; de Jong and van Riel, 2020; Van Limbergen et al., 2020). Furthermore, an experimental study by Torrey et al. (2021) identified genetic strain as a risk factor for mortality rather than the bird's growth rate, indicating an importance of genetic factors for mortality in broilers. It is interesting to note, despite higher age at slaughter, that is, a longer risk period, MAFW is lower in Hubbard compared to Ross. We chose to report mortality risks in this study; using mortality rates would cause the estimated effect of hybrid to be even stronger as this approach also accounts for the longer risk period in Hubbard. The rationale behind reporting mortality risk was to allow for comparison to the vast majority of studies on mortality in broilers, and the all in-all out system meaning populations can be considered closed. The findings of higher mortality on farm in the fast growing Ross, despite less time at risk, is in line with other studies showing poorer health (carcass condemnations, leg health, and dermatitis) in fast growing broilers compared to slower growing broilers (Rayner et al., 2020; Baxter et al., 2021; Forseth et al., 2023), indicating that higher growth rates put more strain on the broiler. These findings are in line with previous research finding lower MFAW in slower growing broiler breeds (Rayner et al., 2020; Baxter et al., 2021). Daily or weekly mortality (as reported by Baxter et al. (2021)), would be preferable as it might be used to assess differences in mortality curves; however, this was not available in the current study.

When assessing the association between hybrid and MAFW we adjusted for seasonal variations and found a higher estimated MAFW in Ross than in Hubbard for all seasons. As for FWM the difference was largest in autumn. In addition, the seasonal variations in mortality were smaller in Hubbard than in Ross. Seasonal variations after the first week are not well described in the existing literature. However, disorders like ascites and sudden death syndrome are known to show seasonal patterns (Julian, 1993; Imaeda, 2000; Forseth et al., 2023). De Jong et al. (2020) suggested that more health problems occur during the more cold and wet seasons, which might be related to suboptimal environmental conditions in the broiler house. Based on previous findings of higher prevalence of ascites in Ross in the same flocks (Forseth et al., 2023), and indications of more ascites in cold climate (Junghans et al., 2022) it could be speculated that the seasonal effect within hybrid on mortality as found here could reflect hybrid differences in disease susceptibility. However, as this analysis was restricted to 1 yr, and the proportion of broilers of each hybrid varied throughout this year, the results can only be suggestive, and not used to conclude on a seasonal effect.

In our study, dead on arrival (DOA) was higher in Ross (0.063%) than in Hubbard (0.015%). The model estimating the association between hybrid and DOA, showed largest estimated difference in DOA under the coldest temperature conditions, with an estimated risk 4.7 times higher for Ross than for Hubbard. Previously reported risk factors for DOA are duration of transport, outdoor temperature, health status and stress or injuries related to catching (Chauvin et al., 2011; Vecerek et al., 2016; Kittelsen et al., 2017). DOA in the present study was low compared to many other European countries (Nijdam et al., 2004; Vecerek et al., 2016; Baxter et al., 2021; Animalia, 2022b). Nevertheless, the transport is without doubt a very stressful situation for the broilers. Chronic diseases present on farm may contribute to DOA as previously described by Kittelsen (2015) who found ascites in 10% of broilers (Ross 308) dead on arrival. The higher prevalence of ascites in fast growing broilers (Baxter et al., 2021; Forseth et al., 2023) might be a main contributor to the observed difference in DOA. Results from the current study indicate hybrid differences in robustness to cope with stress associated with transport to slaughter. Trucks with positive pressure ventilation and heating was prioritized for transports in cold periods, however, type of vehicle was not included in the analysis of our study, neither was transport distance. An effect of cold temperatures on DOA is in line with previous studies (Nijdam et al., 2004). One possible explanation for the more profound effect of low temperatures in Ross might be poorer feather cover (Dixon, 2020; Baxter et al., 2021), which provides less insulation and protection in low temperatures (de Jong et al., 2015).

Previous studies have found a positive association of age and weight at slaughter on DOA (Caffrey et al., 2017); however, when investigating age and weight within hybrid these factors were not statistically significant in this study. Although the Hubbard flocks were slaughtered at a higher age and body weight compared to Ross, the DOA risk in Hubbard was lower than in Ross. In Norway broilers are slaughtered at a relatively low weight. As slaughter weights are often higher in several other countries, this might impact mortality risk both on farm and during transport. High first week mortality is reported as a risk factor both for mortality later in life on farm and for DOA (Kittelsen et al., 2017; Van Limbergen et al., 2020). In the current study, this correlation was found to be negligible for both hybrids. However, it seems clear that knowledge about DOA from studies on fast-growing broilers like Ross 308 cannot necessarily be generalized to other broiler hybrids. In contrast to our findings, Rayner et al. (2020) and Baxter et al. (2021) did not find a difference in DOA between different broiler hybrids. A few extreme values of DOA were excluded from the dataset before analysis (Figure 1). Most of these were caused by accidents. Cases of very high mortalities (also those were mortality was lower than the excluded events) often have different etiology than observations within the normal range (e.g., the IQR) (Kittelsen et al., 2017). The causal pathways behind these events might differ from transports with DOA within normal range. We explored transport records with DOA above the 90th percentile without finding an obvious explanation. However, the vast majority (94%) of the high mortality flocks were Ross. Investigating events of high mortalities might be of interest for welfare as well as economic reasons. On the other hand, many flocks were transported with zero mortalities. For Hubbard, as many as 698/2,395 flocks (29%) had zero mortalities, while the number for Ross was 95/1,809 (5%), indicating that zero mortality transports are indeed possible.

Stockmanship is regarded as the single most important influence of the welfare of farm animals (FAWC, 2007) and lower mortality might be achieved with good farm management. As can be seen in Figure 3, there was a considerable variation in mortality between flocks in all periods of the production cycle, including transport, especially for Ross. As discussed earlier, causes of mortality can be multifactorial, and differences in management might be part of the explanation to the variation between flocks of the same hybrid. Nevertheless, a larger proportion of the unexplained variance across models was found to be between flocks relative to between farms for both hybrids, suggesting that factors varying between flocks had more impact on the unexplained variation than farmer management per se. The findings in the present study suggest that there might be underlying risk factors that also vary between flocks in the same farm, and that mortality in Hubbard broilers compared to Ross might be less affected by external factors, indicating a more robust hybrid.

As this was an observational study, there is always a risk of unmeasured confounding. We cannot exclude that the farmers made other changes in management that might have coincided with the change of hybrid. For the descriptive statistics, the entire study period was included to visualize any long-term trends in on-farm mortality. As can be seen in Figure 3A there is an observed drop in FWM for Ross from 2017 to 2018, which might indicate that there are unknown factors affecting FWM apart from hybrid. For the analyses of FWM and MAFW the study period was limited to the year July 2017 to July 2018 to minimize possible confounding of known temporal changes in hatchery routines. Additionally, only flocks from the same hatchery were included in the study. The present study included both birds that died and culled birds, and the threshold for culling might differ between farmers. In the present study both hybrids were kept by the same farmers thus minimizing the risk of bias caused by different routines for culling. Mortality is easy to recognize, measured in an equal manner in poultry production regardless of country. Nevertheless, an imperfect accuracy of the farmer reported first week mortality might be source of information bias. However, in cases of high FWM, the hatchery will offer economic compensation, under the condition that the farmer reports FWM in due time. This is an incentive for timely and correct registration of FWM. The short study period for FWM and MAFW was a limitation in the present study, in particular for inference of seasonal effects as discussed above. To draw conclusions, a longitudinal study with a study period encompassing several years is needed. As seasonal effects are probably linked to the climatic conditions in the barn, including this information could also be of interest for future studies. Also, more frequent recordings (daily or weekly) of on-farm mortality would be preferable to further investigate broiler mortality and inform preventive measures. For the DOA analysis we utilized data from the full study period. This makes the analysis more sensitive to temporal changes other than the change of hybrid, for example, continued efforts to improve animal health and welfare, as a potential source of bias. To assess this, we performed a sensitivity analysis using only the period of overlap between the hybrids. Overall, the results from the sensitivity analysis showed that for the shorter period the differences in DOA between hybrids were the same, compared to the entire study period.

The validity was deemed good for the source population as few exclusions were made and almost all farmers (93% Figure 1) contracted by Norsk Kylling, consent to participate in the study, thus minimizing the risk of selection bias. Another strength was that the farms included in the study are the same for both hybrids and managed by the same farmers. All broiler flocks were from commercial production; hence, the results are likely generalizable to broilers of the same hybrids kept in similar production systems in temperate areas. No broilers in the present study were necropsied, therefore the underlying etiology behind mortality in the different phases of production could not be determined. Further studies are needed to understand the underlying causal factors for broiler mortality throughout the production period.

In conclusion, we found lower mortalities throughout production, both on farm and during transport, in the slower growing Hubbard JA787 than in the fast-growing Ross 308. Disorders resulting in mortality compromise animal health and welfare, and mortalities late in the production period result in financial losses and affect the sustainability of the value chain. The notable differences in mortality between hybrids are therefore important from multiple perspectives and emphasize the need for more knowledge on causes of mortality in broiler chickens, also considering that there are hybrid differences.

## Appendix Supplementary materials

Image, application 1

## Footnotes

Supplementary material associated with this article can be found in the online version at doi:10.1016/j.psj.2023.103395.
