# Supplementary material for: Mortality risk on farm and during transport: a comparison of 2 broiler hybrids with different growth rates
Source: Poult Sci. 2023 Dec 23;103(3):103395. doi: 10.1016/j.psj.2023.103395 (PMC10825544; doi:10.1016/j.psj.2023.103395)

**Mortality risk on farm and during transport: a** comparison of two broiler hybrids with different growth rates

Merete Forseth^*1^, Randi O. Moe^#^, Käthe Kittelsen^‖^, Ingrid Toftaker^#^

^*^ Norsk Kylling AS, Havneveien 43, 7300 Orkanger, Norway

^#^ Norwegian University of Life Sciences, Faculty of Veterinary Medicine, Elizabeth Stephansens vei 15, 1433 Ås, Norway

^‖^ Animalia, Norwegian Meat and Poultry Research Centre, Lørenveien 38, 0513 Oslo, Norway

^1^ Corresponding author: Merete Forseth, c/o Norsk Kylling AS, Havneveien 43, 7300 Orkanger, Norway, +47 47476020, merete@norsk-kylling.no

Scientific section: Health and disease

**Fig S1.** The study area consisting of two counties in mid Norway. The locations of study farms (n=139) are shown as black dots. (January 1^st^, 2015, to June 22^nd^, 2021

**
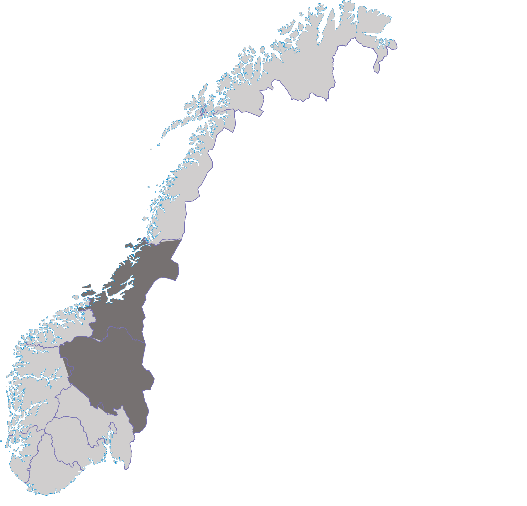
**
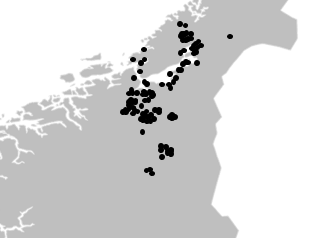

Supplement: Supplementary file 1 [file mmc1.docx]
